# SARS Transmission and Hospital Containment

**DOI:** 10.3201/eid1003.030650

**Published:** 2004-03

**Authors:** Gowri Gopalakrishna, Philip Choo, Yee Sin Leo, Boon Keng Tay, Yean Teng Lim, Ali S. Khan, Chorh Chuan Tan

**Affiliations:** * Ministry of Health, Singapore; † Tan Tock Seng Hospital, Singapore; ‡ Singapore General Hospital, Singapore; § National University Hospital, Singapore; ¶ Centers for Disease Control and Prevention, Atlanta, Georgia, USA; 1 Short-term consultant, Global Outbreak and Alert Response Network (GOARN), World Health Organization

**Keywords:** coronavirus, cross infections, hospital, infection control, nosocomial infections, severe acute respiratory syndrome, Singapore

## Abstract

An outbreak of severe acute respiratory syndrome (SARS) was detected in Singapore at the beginning of March 2003. The outbreak, initiated by a traveler to Hong Kong in late February 2003, led to sequential spread of SARS to three major acute care hospitals in Singapore. The critical factor in containing this outbreak was early detection and complete assessment of movements and follow-up of patients, healthcare workers, and visitors who were contacts. Visitor records were important in helping identify exposed persons who could carry the infection into the community. In the three hospital outbreaks, three different containment strategies were used to contain spread of infection: closing an entire hospital, removing all potentially infected persons to a dedicated SARS hospital, and managing exposed persons in place. On the basis of this experience, if a nosocomial outbreak is detected late, a hospital may need to be closed in order to contain spread of the disease. Outbreaks detected early can be managed by either removing all exposed persons to a designated location or isolating and managing them in place.

Severe acute respiratory syndrome (SARS) has been characterized by efficient transmission in healthcare facilities, highlighting the vulnerability of our modern healthcare system to nosocomial infection (*1*,*2*). Frequent unprotected or inadequately protected patient-to-healthcare worker interactions (*3*) and grouping large numbers of ill persons can greatly amplify intrahospital transmission. If uncontrolled, SARS outbreaks in hospitals may rapidly degrade hospital services and can increase the risk for infection spread into the general community. Hence, rapid and effective containment of hospital SARS outbreaks is important.

In Singapore, an outbreak of SARS was started when a traveler (patient A) visited Hong Kong during February 20 to 25, 2003 (*4*). Patient A returned to Singapore and was admitted to an acute care hospital, Tan Tock Seng Hospital (TTSH), on March 1. Singapore was removed from the World Health Organization’s list of areas with local SARS transmission on May 31. At that time, 206 probable SARS cases had been diagnosed, of which 40.8% were in healthcare workers; 39.8% were in family, friends, or visitors to hospitals; and 12.2% were in inpatients.

The outbreak in TTSH spread to two other tertiary hospitals (Singapore General Hospital [SGH], and National University Hospital [NUH]) despite initial containment efforts (Figure 1). The initial failure of the containment strategy was compounded by the presence, early in the outbreak, of three superspreading events, i.e., SARS patients directly associated with 10 or more secondary infections each. The Table summarizes the profile of the outbreak in the three hospitals. To contain the outbreak in Singapore, we used three separate hospital strategies: 1) closing the hospital, 2) removing an exposed group to a designated hospital, and 3) managing an exposed cohort in place. We review the three hospital containment strategies and the effectiveness of these strategies.

**Figure 1:**
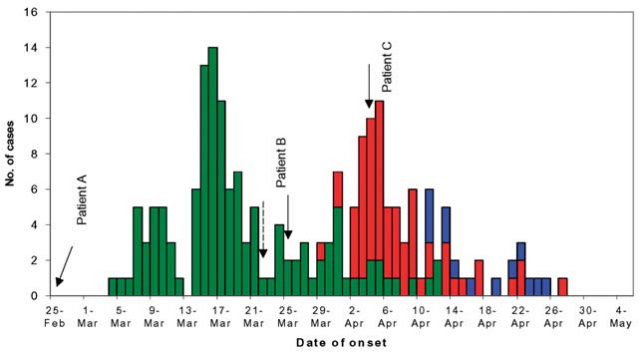
Severe acute respiratory syndrome case-patients infected at three major hospitals, Singapore, February–April 2003. The chart depicts the overall epidemic in each hospital, includes case-patients infected outside the hospital but whose disease origin was linked back to one of the three hospital outbreaks. In Tan Tock Seng Hospital (TTSH), the last case of intrahospital transmission was on April 12. In Singapore General Hospital (SGH), the last case of intrahospital transmission was on April 15. In National University Hospital (NUH), the last case of intrahospital transmission was on April 25. Arrows indicate dates of onsets of the three index cases for each hospital outbreak. Dotted arrow indicates date when full infection control measures (Figure 2) were implemented in TTSH.

**Table:** Key features of the outbreak in Tan Tock Seng Hospital, Singapore General Hospital, and National University Hospital^a^

Features	Tan Tock Seng Hospital (N = 109)	Singapore General Hospital (N = 60)	National University Hospital (N = 10)	
Index case-patient				
Age (y)	22	60	64	
Symptoms/diagnosis	Fever, headache, cough, patchy right infiltrate	Gastrointestinal bleeding, diabetic foot ulcer	Fever, increasing shortness of breath, hypotension	
Time from admission to isolation (d)	6	10	1	
Healthcare workers with probable SARS (%)				
Doctors	8 (7)	3 (5)	1 (10)	
Nurses	35 (32)	21 (35)	2 (20)	
Ancillary caregivers^b^	3 (3)	6 (10)	0	
Others^c^	3 (3)	10 (17)	0	
No. of superspreading events	3 (including patient A)	1	1	
Average time from onset to isolation (d)	4.6	2.6	1	
Age (%)				
<20 y	10 (9)	0 (0)	1 (10)	
20–29 y	40 (37)	7 (11)	4 (40)	
30–39 y	25 (23)	13 (24)	1 (10)	
40–49 y	11 (10)	10 (16)	0	
50–59 y	15 (14)	11 (18)	0	
≥60 y	8 (7)	19 (31)	4 (40)	

^a^SARS, severe acute respiratory syndrome. ^b^Includes technicians, radiographers, and sonographers. ^c^Includes ward clerks, housekeepers, porters, and healthcare attendants.

## Sequential SARS Outbreaks

### TTSH Cluster

The index case, patient A, was admitted to TTSH, a 1,400-bed hospital, on March 1, 2003 for atypical pneumonia. She was treated in a six-bed ward (ward 5A) until she was isolated on March 6.

During the 6 days patient A was in ward 5A, 24 of her primary contacts were infected, and subsequently probable SARS developed in all. These included eight nurses, one health attendant, five patients in the same ward, and 10 visitors.

A second superspreading event occurred when one of the nurses infected by patient A, patient AA, was admitted to an open ward (8A) on March 10. Patient AA was isolated on March 13, by which time 25 persons (12 healthcare workers, four patients in the same ward, eight visitors, and one household contact) had been infected. One of these patients (patient AAA) had multiple medical problems, including diabetes, gram-negative bacteremia, and ischemic heart disease that required her admission to the coronary care unit (CCU) from March 12 to 19. Patient AAA was not isolated for 8 days, resulting in a third superspreading event. Because of her multiple medical problems, SARS infection was not suspected, and healthcare workers caring for her did not use N95 masks. Twenty-seven people at the CCU were infected, including five doctors, 13 nurses, one ultrasonographer, one attendant, two cardiac technicians, and five visitors.

**Figure 2:**
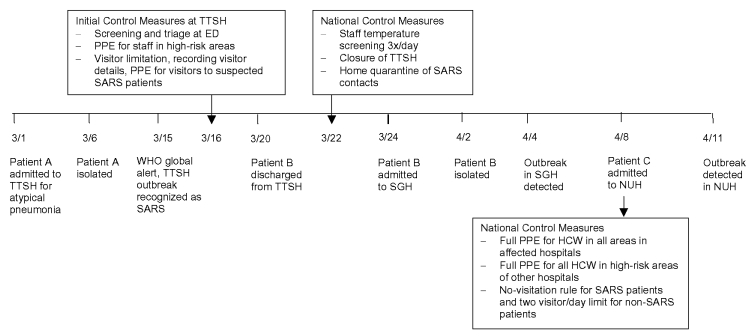
Timeline of events in the outbreak of SARS in the three acute hospitals, Singapore March–May 2003. SARS, severe acute respiratory syndrome; TTSH, Tan Tock Seng Hospital; ED, emergency department; PPE, personal protective equipment (defined as a test-fitted N95 mask, gowns, and gloves; goggles if dealing with suspicious cases; powered air purified respirators for high-risk procedures such as intubation); ICU, intensive care unit; high-risk area defined as ED, ICU, isolation wards; SGH, Singapore General Hospital; NUH, National University Hospital; HCW, healthcare worker. Staff found to have a temperature >37.5°C were given medical leave for 3 days with a review on the third day.

In addition to the spread of SARS within TTSH, infection also spread outside by infected visitors and discharged patients to household contacts and healthcare workers in another hospital, Changi General Hospital. The failure to detect SARS in a discharged patient (patient B) who was a contact of patient A in TTSH led to a second major outbreak in SGH, where this patient was subsequently readmitted (Figure 2).

### Public Health Response

On March 15, probable SARS was diagnosed in a total of 13 persons, all of whom were admitted to TTSH. Six of these case-patients were family members and friends of patient A, six were healthcare workers who had attended to patient A in ward 5A, while one was a healthcare worker from the CCU who had attended to patient AAA. In addition, TTSH was aware of five healthcare workers who were on medical leave for fever, four of whom were from ward 5A and one from ward 8A. Probable SARS subsequently developed in these five healthcare workers.

Probable SARS patients were isolated and their contacts were traced. Symptomatic contacts were isolated while asymptomatic contacts were advised to seek medical attention if they became ill. Infection control precautions were also enhanced in TTSH, including providing separate triage facilities for patients with suspected SARS who may seek treatment at emergency departments and requiring healthcare workers (in the emergency department, intensive-care unit [ICU], isolation wards, triage points, and wards which had been exposed to patients with possible SARS) to wear personal protective equipment consisting of N95 masks, gowns, and gloves. By March 21, approximately 30 probable SARS cases had been reported, of which half were healthcare workers. Persons in at least six wards and the CCU in TTSH had been exposed, but those in additional wards could have been exposed due to movements of infected healthcare workers. Therefore, on March 22, the decision was made to close TTSH’s normal operations and dedicate the hospital solely to treating probable and suspected SARS case-patients (Figure 2). In line with this decision, all healthcare workers in the hospital adopted the use of N95 masks, gowns, and gloves at all times. A strict regimen (3x/day) of temperature surveillance of all staff was also instituted on March 21, with the aim of identifying affected staff as early as possible and isolating them immediately. The hospital limited visitors; visits to SARS case-patients were initially permitted, but visitors were required to use personal protective equipment (i.e., N95 masks, gowns, and gloves). Visitors to other ward areas were asked to use surgical masks. Non-SARS patients were discharged if they had no known exposure to SARS. Figure 2 summarizes the timeline of events in the outbreak and the key control measures instituted.

### Outcome

The intrahospital transmission of SARS in TTSH was controlled within 3 weeks of instituting the measures (Figure 1). In retrospect, if more drastic measures had been implemented on March 15 (when SARS was recognized as the outbreak’s cause), the containment of SARS infection in TTSH and Singapore could have been accelerated. In containing the TTSH outbreak, the inability to rapidly and completely identify all exposed persons was a major problem, mainly due to the absence of visitor records and the high frequency and complexity of patient-healthcare worker contacts and movements. Healthcare workers were also not using full personal protective equipment for most of that time.

If all contacts of the initial SARS case-patients detected on March 15 had been identified, patient AAA could have been cared for in isolation in CCU by staff with full personal protective equipment. At least 22 of the 27 case-patients infected by patient AAA in the CCU possibly could have been prevented.

The discharge of patient B from the same ward in TTSH as patient A was another factor which led infection to spread to SGH. Patient B had not been identified as a contact of patient A at that time and was subsequently readmitted to an open ward in SGH. In retrospect, we think that if all discharges from affected areas in TTSH had been stopped on March 15, the 60 probable SARS cases in SGH linked to patient B might have been prevented.

In-depth interviews to determine the epidemiologic link of SARS case-patients that arose after March 15 indicated that at least 17 of them visited TTSH areas where initial cases arose. If a no-visitor policy had been implemented in TTSH on March 15, at least 17 new SARS cases outside of TTSH could theoretically have been prevented.

### SGH Cluster

The index case-patient in SGH was patient B, who did not exhibit typical signs and symptoms of SARS. Patient B was admitted to an open ward, ward 57, in SGH on March 24 for gastrointestinal bleeding. A fever attributed to an *Escherichia coli* urinary tract infection developed on March 26. On March 29, he was transferred to an adjacent open ward, ward 58, where he remained until April 2. He was transferred to the diagnostic radiology department twice, on April 1 and 2, respectively. Results of repeated chest x-rays were normal until shadowing in the right lower zone and left perihilar region was noted on April 5.

The SGH outbreak was identified on April 4 (Figure 2) when fever developed in a cluster of 13 healthcare workers. The cluster (one doctor, 11 nurses, and one radiographer) was detected because temperatures of all healthcare workers in SGH were monitored 3x/day. All 13 healthcare workers had attended to patient B; probable SARS developed in all 13. In total, the SGH outbreak resulted in 60 (24 healthcare workers, 11 inpatients, two outpatients, 12 visitors and 11 household contacts) probable SARS cases.

### Public Health Response

The public health response was based on two key considerations. First, with 1,600 beds, SGH is the largest acute hospital in Singapore. With TTSH, the second largest acute hospital, dedicated as a SARS hospital, authorities could not shut down SGH as well. Second, the epidemiologic assessment was that the outbreak was localized. All SARS cases came from wards 57 and 58. Symptom onset for the 13 affected healthcare workers was March 31 for one case-patient, April 2 for three case-patients, April 3 for four case-patients, and April 4 for five case-patients. Most importantly, in line with SGH’s standing rule, no affected healthcare workers came to work when they had fever. There was no evidence of secondary transmission.

The key containment strategy in the SGH was to completely remove exposed patients and healthcare workers to TTSH, the designated SARS hospital. Three main groups of patients were identified who might have been exposed to SARS in wards 57 and 58 during the “hot” period (i.e., when patient B was admitted on March 24 to the time his contacts were identified and transferred to TTSH on April 5).

The first group identified was the 80 patients in wards 57 and 58 on April 5. Next identified were a total of 135 patients admitted to the two affected wards during the hot period and subsequently transferred to three other wards in SGH. These three wards were subjected to a no-admission, no-discharge policy, and the two groups of patients were transferred to TTSH. The third group was 386 patients who had been in wards 57 and 58 in the hot period but had been discharged. None of these patients had fever, and all were called by phone 3x/day for 10 days.

Medical staff members in wards 57 and 58 were sent to TTSH to care for the transferred patients. An additional 236 SGH healthcare workers who had been in contact with exposed healthcare workers from the two wards were quarantined for 10 days.

### Outcome

Containment measures controlled intrahospital infection spread within 10 days. Of the exposed patients and healthcare workers transferred to TTSH, probable SARS developed in eight. All were infected before the transfer.

However, two shortcomings affected the containment strategy. First was the failure to fully trace back the index case-patient’s (patient B’s) movement in the hospital. As a result, two exposed clinical areas were missed in the initial containment strategy. These areas were recognized when 11 additional cases were detected in two new areas in SGH: the diagnostic radiology department and a tertiary cancer facility, National Cancer Centre (NCC). Patient B had been in the diagnostic radiology department on April 1, where two healthcare assistants, two outpatients, and one visitor were infected. During patient B’s second visit to the radiology department on April 2, an additional two healthcare workers who attended to him were infected. Fortunately, the cases were detected early because of SGH’s strict 3x/day temperature monitoring regimen for all staff. In addition, all healthcare workers had begun using test-fitted N95 masks in all settings, including staff meetings and briefings. These steps helped mitigate further transmission of infection by healthcare workers. Unfortunately, one healthcare worker in the radiology area was given medical leave when she became febrile. She had close contact with some friends while on medical leave, leading to infection in four of them, one of whom infected four family members.

The second, more serious shortcoming arose because visitors to the two affected wards during the hot period were not completely traced. Patient B was visited by his brother, patient C, at SGH on March 31. Eight days after the visit, symptoms of SARS developed in patient C, who was admitted to an open ward in NUH.

### NUH Cluster

Patient C sought treatment at the emergency department of NUH on April 8 (Figure 2). He remained in the emergency department for approximately 4 hours before being transferred to an open ward, ward 64, where he remained for about 8 hours before being intubated and transferred to the ICU. He remained isolated in the ICU until the morning of April 9 when he was transferred to TTSH; probable SARS was diagnosed after a complete history of contact was elicited.

### Public Health Response

The NUH outbreak was identified on April 11 when a doctor who had attended to patient C was noted to have fever. The key containment strategy in the NUH outbreak was isolating exposed persons (closing the exposed ward, stopping all new admissions and discharges to the ward for a period of 10 days, and isolating exposed patients). Staff members were put on work quarantine (i.e., they continued to work but were quarantined after work in separate quarters) and 3x/day temperature monitoring.

### Outcome

As a result of these measures, when fever developed in two nurses 2 days after the outbreak, they were quickly recognized and isolated the same day. Fever developed in five inpatients in ward 64, who were transferred to TTSH. Two of these five inpatients had direct contact with patient B, and infections in the other three were likely acquired either from one of the infected healthcare workers or inpatients. The last probable SARS case from this ward was detected on April 25.

The shortcoming in this containment strategy was the failure to identify all the exposed visitors, one of whom had come into contact with patient C on April 8. This visitor was unidentified until probable SARS developed on April 11, by which time two family contacts had been infected.

## Discussion

In the sequential SARS outbreaks, the three acute hospitals used different key containment strategies. On the basis of this experience, three key factors must be considered when deciding on the appropriate containment response. First, has the outbreak has been detected early? Second, can the likely source of the infection cluster be rapidly identified? Third, can a complete list of all contacts be obtained within 48 hours?

In our experience, if an outbreak is recognized early (within one incubation period and no evidence of secondary transmission on careful contact tracing) and the source identified and isolated expeditiously, the outbreak can probably be contained by closing the ward or clinical area, isolating all patients in the ward, and quarantining healthcare workers and visitors who have been in the ward. The NUH outbreak focused on this as its central containment strategy. The key was identifying and isolating patient C early. Patient C was in NUH for ≤12 hours before being isolated; hence, the number of exposed persons was manageable, and contacts could be identified with a high degree of confidence.

If, however, the outbreak is detected late (i.e., beyond 1–2 incubation periods, when secondary cases have occurred among staff and inpatients and multiple wards have been exposed), the most prudent course in our view is to close the hospital and place all healthcare workers on work quarantine as an immediate measure, while conducting epidemiologic investigations and implementing containment measures. We would generally consider an outbreak detected late if >1 maximum incubation period (10 days) elapsed before the outbreak was recognized. For example, the outbreak in TTSH was detected 14 days after the admission of patient A. In retrospect, by this time, secondary transmission in different parts of the hospital was indicated by the fact that SARS was diagnosed in a household contact of patient AA and a healthcare worker who attended to patient AAA in the CCU.

All patient admissions and discharges should be stopped. A complete list of all SARS patient contacts needs to be generated within 48 hours and those contacts quarantined or kept under medical surveillance, e.g., 3x/day telephone calls by a nurse. Otherwise SARS infection could rapidly spread to other healthcare facilities.

If closing the whole hospital is not possible, we believe that transferring all exposed persons to a designated SARS facility is the next best option. We have observed that running normal hospital services is difficult while managing a large number of patients with SARS or exposure to SARS. Scarcity of available beds may lead to transferring exposed patients prematurely out of isolation facilities into general wards, risking continued infection transmission. In addition, transferring all exposed persons out of the affected hospital allows it to carry on normal services as much as possible.

This containment strategy was adopted for the SGH outbreak and resulted in controlling intrahospital infection spread within 10 days. Movements of the index case-patient should be investigated to ensure that all exposed persons are quarantined. In the SGH outbreak, failing to identify the NCC and radiology departments as exposed areas led to missing exposed healthcare workers and patient contacts, and a small cluster of 10 secondary cases arose as a result.

### Contact History

Patients’ contact history must be reviewed carefully for contact with SARS patients. Because the contact history for both patients B and C was not thoroughly obtained at admission, they were not isolated, resulting in the SARS outbreaks in SGH and NUH, respectively.

### Fever Surveillance of Healthcare Workers

In Singapore, all hospitals were required to establish a system of thrice-daily temperature surveillance of staff. In hospitals affected by SARS, temperatures were checked by designated staff and recorded. In other hospitals, staff could check and report their own temperatures. Temperature surveillance covered all healthcare workers, including healthcare attendants, cleaners, and contract staff who worked in clinical areas. Healthcare workers were not allowed to work if their temperature was >37.5°C, and any healthcare worker with fever for >3 days or occurring as part of a cluster of cases was isolated. When more than two staff members or patients in a clinical area were febrile, epidemiologic investigation was initiated.

In our experience, fever surveillance among healthcare workers rapidly identified potentially infected healthcare workers in hospitals with SARS outbreaks. In the SGH outbreak, fever surveillance enabled the rapid detection of 11 probable cases that arose from the NCC and radiology departments. Similarly in the NUH outbreak, temperature surveillance identified two ward 64 nurses who were isolated the same day.

### Personal Protective Equipment

On the basis of our and others’ experiences (*5*), the nonspecific symptoms of SARS make identifying potential cases difficult. Strictly adhering to the use of personal protective equipment by healthcare workers mitigated spread in all three outbreaks.

### Visitor Records

In all three hospital outbreaks, visitors contributed substantially to SARS transmission. At least 21 cases resulted from spread by hospital visitors to family and community contacts. This type of transmission emphasizes the importance of maintaining a visitor log and limiting visitors. In Singapore, a no-visitor policy in all public hospitals was implemented 2 weeks after detecting the NUH outbreak. Similar restrictions on hospital visitors were implemented as control measures in outbreaks elsewhere (*5*).

### Better Preparedness

The outbreak in TTSH was central to spreading infection to the other two hospitals. Key difficulties in containing the TTSH outbreak were: 1) late recognition of disease, 2) lack of understanding of the disease, 3) inadequate infrastructure to support outbreak management of this scale, 4) lack of ability to identify atypical cases, and 5) lack of understanding of superspreading events.

Some of these difficulties can be overcome by better planning and preparedness. However, a large part of an effective response will rely on thoroughly understanding the disease. In Singapore, we tried to develop a better response capability through several means. The first component was prevention through the use of N95 masks, gowns, and gloves by healthcare workers in high-risk clinical areas and triaging febrile patients at emergency departments and outpatient clinics, followed by isolating infectious patients early. The second component was detecting possible SARS clusters early through surveillance for clusters of febrile healthcare workers or patients. The third component was ensuring that all hospitals have established and tested systems to rapidly generate a complete list of all potential healthcare workers, patients, and visitor contacts. These components should be part of a hospital preparedness plan.
